# Supplementary material for: Simphony: simulating large-scale, rhythmic data
Source: PeerJ. 2019 May 23;7:e6985. doi: 10.7717/peerj.6985 (PMC6535214; doi:10.7717/peerj.6985)
Supplement: Supplemental Information 1 [file peerj-07-6985-s001.pdf]

# Supplemental Figures for:

# Simphony: simulating large-scale, rhythmic data

Jordan M. Singer[1], Darwin Y. Fu[1], Jacob J. Hughey[1,2,*]

[1]Department of Biomedical Informatics, Vanderbilt University Medical Center, Nashville, Tennessee; [2]Department of Biological Sciences, Vanderbilt University, Nashville, Tennessee

*To whom all correspondence should be addressed: jakejhughey@gmail.com

Figure S1

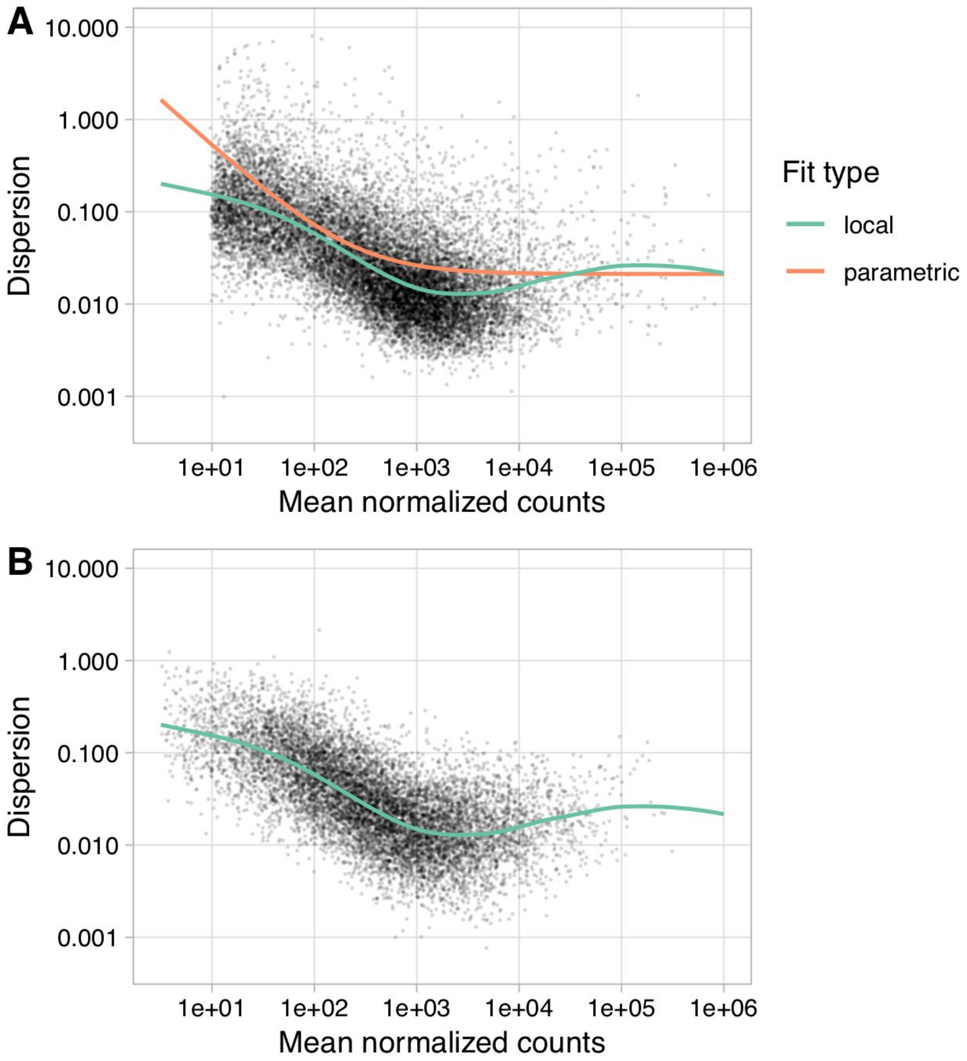


Estimating distributions of mean and dispersion and the relationship between the two. **(A)** Scatterplot of gene-wise means and dispersions in RNA-seq data from mouse liver (PRJNA297287), along with parametric and local regression-based curves, calculated using DESeq2. The curve based on local regression was chosen as the default in Simphony. **(B)** Scatterplot of sampled means and dispersions (n = 10,000), with values drawn from the estimated distributions of mean normalized counts and residual log dispersion (around the local regression-based curve).

Figure S2

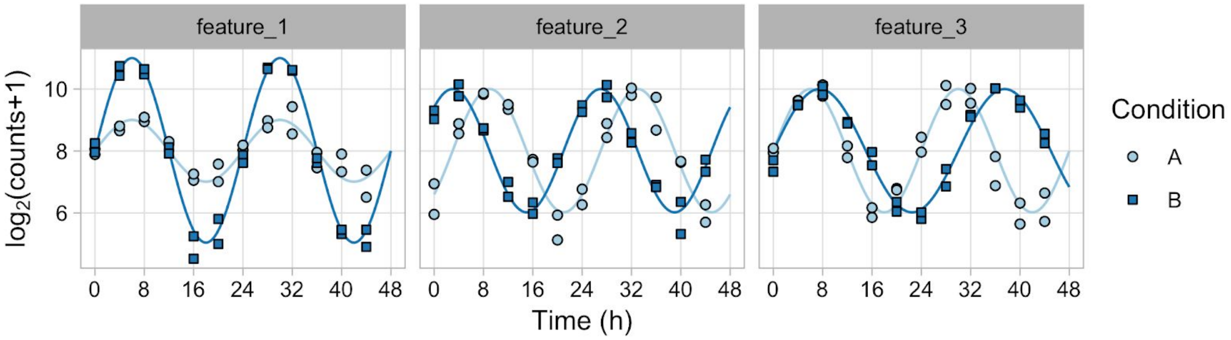


Examples of differentially rhythmic gene expression data generated by Simphony. Gene expression values were sampled from the negative binomial family. Rhythms followed a sinusoid. The baseline $\log_2$ counts was 8. Time points in each of two conditions were spaced 2 h apart, with 2 replicates per time point. Colored circles and squares show the sampled gene expression values in each condition. Colored lines show the expected expression over time in each condition (feature_1 shows a difference in amplitude, feature_2 shows a difference in phase, feature_3 shows a difference in period).

Figure S3

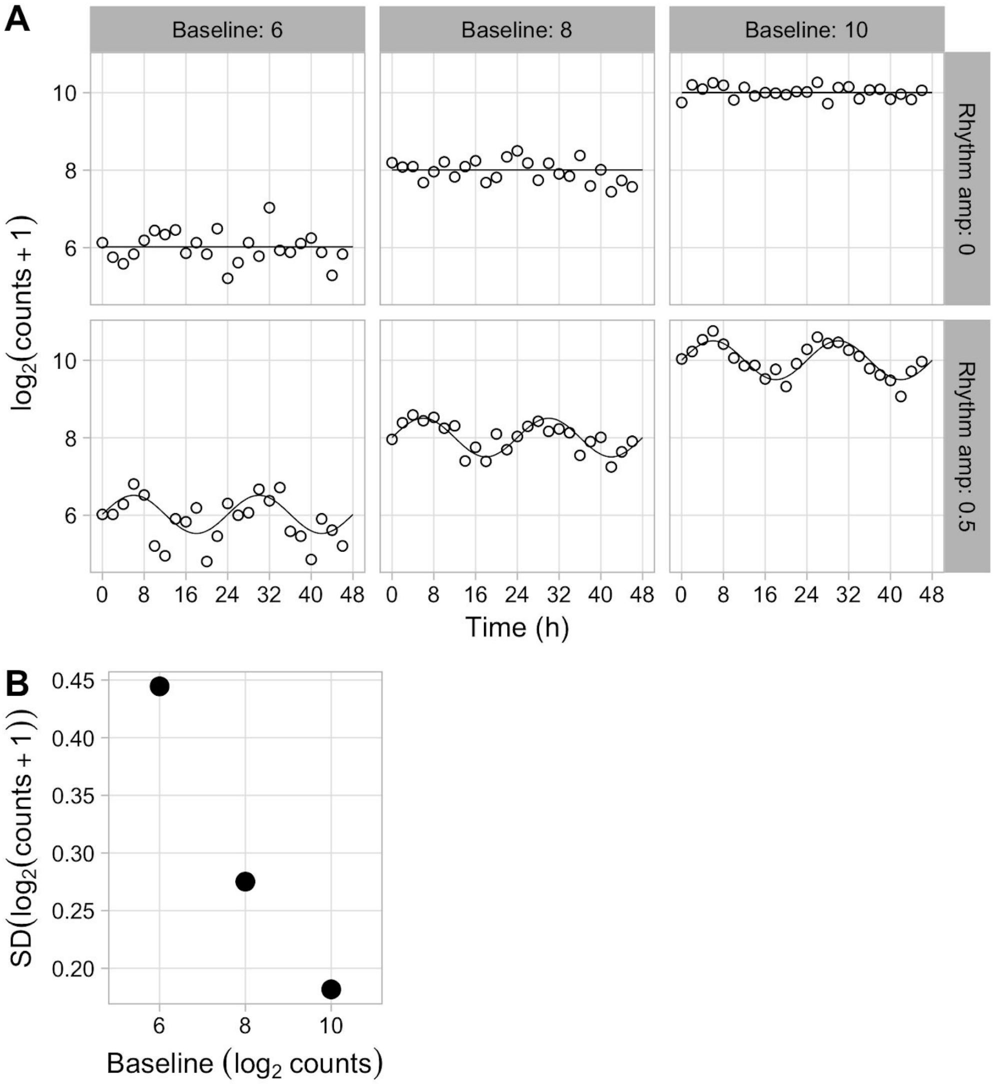


Examples of non-rhythmic and rhythmic gene expression data generated by Simphony, with various values of baseline expression and rhythm amplitude. The interval between time points was 2 h, with 1 replicate per time point. **(A)** Each plot shows the time-course for one simulated gene. Circles show the sampled expression values (from the negative binomial family) and black lines show the expected expression over time. **(B)** Standard deviation of $\log_2$(counts+1) for non-rhythmic genes for each value of baseline expression.
